# Virulence gene profile and antimicrobial resistance patterns of *Aeromonas hydrophila* in farmed catfishes (*Heteropneustes fossilis* and *Pangasianodon hypophthalmus*) for the first time in Bangladesh

**DOI:** 10.1371/journal.pone.0331943

**Published:** 2025-09-12

**Authors:** Farjana Ferdousi, Bushra Benta Rahman Prapti, Alamgir Hasan, Aminur Rahman, Ravi Yadav, Md. Shafiqul Islam, Md. Taohidul Islam, Md. Alimul Islam, Mahbubul Pratik Siddique

**Affiliations:** 1 Department of Microbiology and Hygiene, Faculty of Veterinary Science, Bangladesh Agricultural University, Mymensingh, Bangladesh; 2 Department of Medicine, Faculty of Veterinary Science, Bangladesh Agricultural University, Mymensingh, Bangladesh; Mansoura University, EGYPT

## Abstract

Among several *Aeromonas* spp., causing diseases in fishes, *Aeromonas hydrophila* is the most predominant and pathogenic one. This study represents the first molecular detection, virulence profiling, and antibiogram analysis of *Aeromonas hydrophila* isolated from stinging catfishes (shing; *Heteropneustes fossilis*) and shark catfishes (pangasius; *Pangasianodon hypophthalmus*) in Bangladesh. Whole fish samples (n = 140) were collected from fish farming areas of Trishal upazila (stinging catfish 50; shark catfish 20) and Muktagachha upazila (stinging catfish 50; shark catfish 20), under Mymensingh district. Isolation and identification were accomplished through cultural, morphological, biochemical and finally, polymerase chain reaction (PCR) using genus- and species-specific primers, targeting *16S rDNA* gene. Out of 140 samples, 38 (27.14%) isolates were found positive for *A. hydrophila* via conventional and PCR. The PCR-based virulence profiling showed that *aer*A (44.73%), *hly*A (39.74%), *asa*1 (39.47%), *ahy*B (60.52%), *act* (21.5%), *ast* (28.94), *alt* (47.36%), *ser* (44.73%), *lip* (50%), *gcat* (23.68%), and *asc*V (21.05%) genes were detected, however, no *aex*T was detected. Antibiotic susceptibility test (using disc diffusion method) revealed that highest resistance (other than Penicillin Group) against Aztreonam and Cefuroxime (73.68%), and lowest against Gentamicin and Azithromycin (5.26%). MDR was detected in 71.05% isolates, and 92.11% isolates had MAR index ≥ 0.2. Statistically significant associations were observed between phenotypic resistance and specific virulence genes p ≤ 0.05. It could be concluded that MDR and virulent potential *A. hydrophila* are prevalent in stinging and shark catfishes of Mymensingh region, which might be a serious threat to sustainable aquaculture, food safety and public health.

## Introduction

Aquaculture sector is a booming sector, ensuring global food security, with supply of nutrition (relatively cheaper but high-quality proteins; 60% of total protein intake globally), and substantiate financial gain and employment opportunities [1]. Bangladesh, with her world’s largest flooded wetland, has emerged as one of the supreme fish-producing countries (global ranking in 2018: inland fish production- 3^rd^; aquaculture production- 5^th^; marine fish production- 11^th^) [2], considered as major protein source [3] and sustainable export sectors [4]. In Bangladesh, freshwater aquaculture predominantly utilizes pond-based farming of carp (both indigenous and exotic), catfishes [stinging catfish (*Heteropneustes fossilis*) and shark catfish or pangasius (*Pangasianodon hypophthalmus*)], and few small indigenous fish (SIF), particularly, tilapia (*Oreochromis mossambicus*), and others, such as, punti (*Puntius ticto*), walking catfish (*Clarias batrachus*), climbing perches (*Anabas testudineus*), and shorpunti (*Puntius sarana*) [2]. Among them, the stinging catfish (*H. fossilis*; local name: shing) is one of the most familiar and both economically and nutritionally valuable fish in Bangladesh to almost all ages of people [5]. In many parts of the Indian subcontinent, it is considered particularly nutritious, tasty, and digestible, with less spine, fat, and digestibility than animal fats and it has nutritional (rich in iron and calcium content) and medicinal value (advised in anaemia, weakness) [6]. Besides that, shark catfish or pangasius (*Pangasianodon hypophthalmus*) is one of the most important and popular species in aquaculture of Bangladesh for its rapid growth, high productivity, tolerable cost and supplementation to the local market [7]. However, in commercial fish farms, bacterial infections are the most common infectious cause of significant mortality [8]. Among other bacterial pathogens, *Aeromonas* spp. are considered as the most dominant and leading bacterial pathogens in farmed fish [9].

*Aeromonas hydrophila* (*A. hydrophila*) is an opportunistic but major pathogen of fish [10], also associated with human diseases, causes skin and soft tissue infection, gastroenteritis, urinary tract infection, pneumonia, and necrotizing fasciitis [11–13], and distributed ubiquitously in soil and all sorts of water bodies [14,15]. It is a Gram-negative, non-spore forming, motile bacterium under the *Aeromonadaceae* family, causes diseases including haemorrhagic septicaemia, exophthalmia, abdominal distortion, motile aeromonas septicaemia (MAS) and so on to aquatic species [11]. Motile aeromonads are abundant in freshwater settings all over the world [16], and their high mortality rates in the aquaculture industry regularly cause large financial losses [17].

Virulence factors, most importantly present in *A. hydrophila*, assist causing pathogenicity to the fishes and also in other species. Bacterial pathogens are judged on their virulence factors and toxicity utilizing virulence factors [18] and their combined effect contributes to *A. hydrophila* pathogenicity [17,19,20]. *Aeromonas* virulence is multifactorial, allowing the bacteria to populate, infiltrate, and conquer the host’s immune response, resulting in an infection that causes disease [21,22]. It had been well documented that the magnitude of clinical manifestations, during disease episode, clearly resulted from the impacts of several virulence factors of *A. hydrophila* [23]. Among numerous virulence associated factors, the most commonly studied are the adhesins (heat-labile enterotoxins (*alt*), elastase gene (*ahy*B)), toxins (aerolysin (*aer*A), haemolysin (*hly*A), cytotoxic enterotoxin (*act*)), type III secretion system (structural gene (*asc*V**),** T3SS effector protein (*aex*T)) and enzymes used for invasion and degradation of host tissue (lipase (*lip*), *ser* (serine protease), cytotoxic heat-stable enterotoxins (*ast*)), strengthening the pathobiologic mechanism of *A. hydrophila* [9,16,24]. To understand the epidemiologic features and exact pathobiologic mechanism, the investigation of *A. hydrophila* virulence factors is a critical issue [23]. However, there is a noticeable gap in studies exploring the distribution and function of virulence genes in *Aeromonas* spp. isolated from clinically infected fish, particularly in aquaculture settings [25]. In the context of Bangladesh, the present research work could be considered as the pioneer work regarding the virulence gene detection in *A. hydrophila* from stinging catfish and shark catfish.

Furthermore, the increased use of antimicrobial agents in clinical outbreaks of various diseases of aquatic species have been contributing in accelerated tolerance of *Aeromonas* species isolated from aquatic ecosystems [12], and subsequently, accumulation of antimicrobial residues both in the aquaculture products and the environment are getting worse [24]. Multiple antimicrobial/antibiotic resistance (MAR) from *A. hydrophila* is a worldwide problem [26], due to the presence of mobile genetic elements (MGEs; *viz*., plasmids, transposons, integrons) in both environmental and clinical isolates [27,28]. Contamination of the environment is thought to be the most efficient way to select resistant populations and exchange resistant genes [12,26,28]. In fact, *Aeromonas* has already been proposed as model genus for profiling waterborne antimicrobial resistance genes (ARGs) globally due to its ubiquity, clinical relevance, ARG diversity, role in gene transfer, ease of study, and sensitivity to human-driven environmental changes [27]. In Bangladesh, antibiotic use was reported by 22 farms (3%) within the 24 hours prior to the interview, 36 farms (5%) within the past 72 hours, 141 farms (21%) within the past 14 days, and 478 farms (71%, 68–75% CI) at least once since the beginning of their production cycle. The prevalence of antibiotic use in the 14 days leading up to the interviews was significantly higher among freshwater fish farms (98%) compared to brackish water farms (2%). The antibiotics most commonly used included oxytetracycline, ciprofloxacin, and amoxicillin. These medications were primarily administered for both treatment and disease prevention purposes [29].

Despite Bangladesh’s rapid advancement in aquaculture production, there remain critical knowledge gaps, especially concerning disease outbreaks in farmed fish, the molecular characterization of fish pathogens, and the monitoring of antimicrobial resistance (AMR) patterns. Surveillance of pathogenic bacteria like *A. hydrophila*, especially in economically significant fish species such as stinging catfish (shing) and shark catfish (pangasius), is often limited to phenotypic identification, without delving into virulence gene profiling or molecular resistance mechanisms [30].

From these perspectives, in this study, first we isolate and identify *A. hydrophila* at molecular level from stinging catfish and shark catfish using routine bacteriological methods and polymerase chain reaction (PCR) assays, then investigated numerous virulence associated genes using PCR assay and finally, determined the antibiotic susceptibility by disc diffusion method.

## Materials and methods

### Ethical approval

This study got approval from the Animal Welfare and Experimentation Ethics Committee (AWEEC), Bangladesh Agricultural University (BAU), Mymensingh-2202, Bangladesh [(AWEEC/BAU/2020(3)].

### Sampling and sampling areas

**Fig 1:**
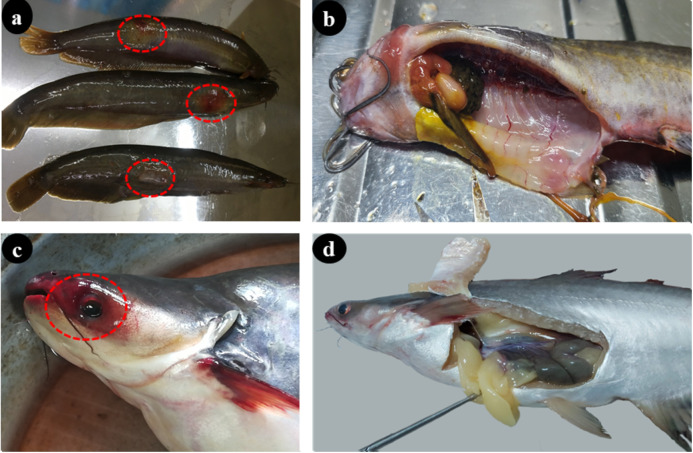
Sample collection and processing from suspected *Aeromonas* infected cat fishes. (a) stinging cat fish samples, where circle indicated infected skin (ulceration and haemorrhage); (b) collection of Intestine (haemorrhagic) and Gill (normal) of stinging cat fish; (c) shark cat fish sample, circle indicated lesion on head region; and (d) collection of intestine and gill (normal appearance) of shark cat fish.

Whole fish samples (n = 140), which showed clinical signs of suspected *Aeromonas* infection, were collected from fish farming areas, comprised of 50 stinging catfish and 20 shark catfish from Trishal upazila (sub-district) and 50 stinging catfish and shark catfish from Muktagachha upazila. Though the fishes were alive at the time of collection, they naturally died during transportation in icebox (0–4°C) before arrival at the laboratory. To ensure death and eliminate possible residual sufferings, fish were humanely sacrificed by pithing prior to dissection. No anaesthesia or analgesia was required, as fish were not subjected to painful manipulations before sacrifice. All possible efforts were made to minimize suffering, including rapid transport, maintenance of cool conditions, and immediate post-mortem processing. From each fish, the skin, gill, and intestine samples have been taken (Fig 1) and examined for bacteriological study, virulence genes, and antibiogram study. Microbiological study was performed in the Department of Microbiology and Hygiene, BAU, Mymensingh-2202, Bangladesh.

### Bacterial isolates

The suspected skin, gill and intestine of fishes were collected in sterile condition and seeded on Alkaline Peptone Water (APW) for primary enrichment and one loop full of enriched sample was streaked on Trypticase soy agar (TSA) (Hi-Media, India) plates, followed by incubation at 37°C for 24 hr. In addition, suspected single colony was sub-cultured on Thiosulfate-citrate-bile salt-sucrose agar (TCBS) (Hi-Media, India), which act as a selective media for *A. hydorphila* for 24 hr at 37$\backslash {(}^{\circ }\text{C}$. The 5% defibrinated sheep blood agar with ampicillin (0.002 g) medium was prepared for detection of haemolysis activity of *A. hydrophila* according to the method of Wang et al. [31]. The motility test was performed by MIU (Motility Indole Urease) medium (Hi-Media, India) for the confirmation of motility of *A. hydrophila*. Furthermore, the Gram staining and biochemical tests including Catalase, Voges-Proskauer (VP) test, Methyl-red (MR) test, Indole test and sugar fermentation test such as Dextrose, Maltose, Sucrose, Mannitol, and lactose fermentation were performed. Eventually, suspected colonies with typical morphology were selected and identified through polymerase chain reaction (PCR) assay using genus-specific primers *16S rDNA* (F-5’CTACTTTTGCCGGCG3’ and R-5’TGATTCCCGAAGGCA3’) by Lee et al. [32] and *16S rDNA A. hydrophila* species-specific primers (F-5’GAAAGGTTGATGCCTAATACGTA3’ and R-5’CGTGCTGGCAACAAAGGACAG3’) according to Gordon et al. [33].

### Genomic DNA extraction

The DNA of the isolates were extracted by boiling and thawing method according to the procedure of Siddique et al. [34] with little modification. In brief, 1 ml seeded broth was taken in a Eppendorf tube and centrifuged at 1000 rpm for 3 minutes, then the supernatant was discarded and 200 μl distilled water was added to the remaining pellet. The mixture was vortexed and kept into the ice for ice shock after being boiled in hot water for 10 minutes and finally centrifuged at 1000 rpm for 3 minutes. An amount of 100 μl of supernatant was collected in another sterile Eppendorf for PCR amplification as template DNA. The quantity and quality of DNA was measured by Nanodrop One (Thermo Fisher Scientific^TM^, USA).

### PCR amplification of genus- and species-specific *16S rDNA* gene

The ribosomal *16S rDNA* gene, both for genus and species specificity, was amplified in PCR reactions using the mentioned primers and the total volume of PCR reactions mixture was 25 μl including 12.5 μl PCR Master Mix, 2X (Promega, USA), 1 μl forward primer, 1 μl reverse primer, 5 μl DNA template, and 5.5 μl nuclease free water. For the PCR amplification of genus-specific gene, the thermal program was: 1 cycle of initial denaturation at 94°C for 4 min, then 35 cycles of denaturation at 94°C for 1 min, annealing at 68°C for 30 sec, extension at 72°C for 45 sec, and 1 cycle of final extension at 72°C for 10 min [32]. For species-specific gene amplification through PCR, the thermal program was: 1 cycle of initial denaturation at 94°C for 3 min, then 30 cycles of denaturation at 94°C for 30 sec, annealing at 55°C for 40 sec, extension at 72°C for 30 sec, followed by 1 cycle of final extension at 72°C for 10 min [33]. The PCR products were analysed on 2% agarose gel stained in ethidium bromide, and finally visualized and documented using UVsolo TS Imaging System (Biometra GmbH, Rudolf-Wissell-Str. 30, Lower Saxony, Goettingen, D-37079, Germany).

### Detection of virulence genes

All positive strains were subjected to PCR assays to detect the twelve (12) virulence genes (*aer*A*, alt, ahy*B*, asa*1*, ast, act, hly*A*, aex*T*, asc*V*, lip, ser,* and *gca*t) using the primers and conditions described by previous researchers, mentioned in the Table 1.

**Table 1:** Primers used for the PCR amplification of virulence genes in *A. hydrophila* isolates.

Target gene	Primer sequence (5’to 3’)	Amplicon size	Thermal conditions	References
*aer*A	F: CCTATGGCCTGAGCGAGAAG	431 bp	Initial Denaturation-94$\backslash {(}^{\circ }\text{C}$ 2m, Denaturation-94$\backslash {(}^{\circ }\text{C}$ 30s, Annealing-62$\backslash {(}^{\circ }\text{C }$50s, Extension-72$\backslash {(}^{\circ }\text{C}$ 30s; cycle 35 and Final extension-72$\backslash {(}^{\circ }\text{C}$ 10m.	[35]
	R: CCAGTTCCAGTCCCACCACT			
*alt*	F: TGACCCAGTCCTGGCACGGC	442 bp	Initial Denaturation-94$\backslash {(}^{\circ }\text{C}$ 5m, Denaturation-94$\backslash {(}^{\circ }\text{C}$ 30s, Annealing-59$\backslash {(}^{\circ }\text{C }$30s, Extension-72$\backslash {(}^{\circ }\text{C}$ 1m; cycle 30 and Final extension-72$\backslash {(}^{\circ }\text{C}$ 10m.	[36]
	R: GGTGATCGATCACCACCAGC			
*ahy*B	F: ACACGGTCAAGGAGATCAAC	513 bp	Initial Denaturation-95$\backslash {(}^{\circ }\text{C}$ 10m, Denaturation-95$\backslash {(}^{\circ }\text{C}$ 15s, Annealing-66$\backslash {(}^{\circ }\text{C }$30s, Extension-72$\backslash {(}^{\circ }\text{C}$ 30s; cycle 25, and Final extension-72$\backslash {(}^{\circ }\text{C}$ 10m.	[36]
	R: CGCTGGTGTTGGCCAGCAGG			
*asa*1	F: TAAAGGGAAATAATGACGGCG	249 bp	Initial Denaturation-95$\backslash {(}^{\circ }\text{C}$ 5m, Denaturation-95$\backslash {(}^{\circ }\text{C}$ 30s, Annealing-59$\backslash {(}^{\circ }\text{C }$30s, Extension-72$\backslash {(}^{\circ }\text{C}$ 30s; cycle 35, and Final extension-72$\backslash {(}^{\circ }\text{C}$ 7m.	[31]
	R: GGCTGTAGGTATCGGTTTTCG			
*ast*	F: TCTCCATGCTTCCCTTCCACT	331 bp	Initial Denaturation-95$\backslash {(}^{\circ }\text{C}$ 5m, Denaturation-95$\backslash {(}^{\circ }\text{C}$ 1m, Annealing-55$\backslash {(}^{\circ }\text{C }$1m, Extension-72$\backslash {(}^{\circ }\text{C}$ 1m; cycle 30, and Final extension-72$\backslash {(}^{\circ }\text{C}$ 5m.	[36]
	R: GTGTAGGGATTGAAGAAGCCG			
*act*	F: GAGAAGGTGACCACCAAGAACAA	232 bp	Initial Denaturation-94$\backslash {(}^{\circ }\text{C}$ 5m, Denaturation-94$\backslash {(}^{\circ }\text{C}$ 30s, Annealing-56$\backslash {(}^{\circ }\text{C }2m$, Extension-72$\backslash {(}^{\circ }\text{C}$ 1m; cycle 30, and Final extension-72$\backslash {(}^{\circ }\text{C}$ 5m. (slightly modified0	[37]
	R: AACTGACATCGGCCTTGAACT			
*hly*A	F: GCCGGTGGCCCGAAGATACGGG	597 bp	Initial Denaturation-94$\backslash {(}^{\circ }\text{C}$ 5m, Denaturation-94$\backslash {(}^{\circ }\text{C}$ 30s, Annealing-62$\backslash {(}^{\circ }\text{C }$30s, Extension-72$\backslash {(}^{\circ }\text{C}$ 2m; cycle 30, and Final extension-72$\backslash {(}^{\circ }\text{C}$ 10m	[38]
	R: GGCGGCGCCGGACGAGACGGG			
*aex*T	GGCGCTTGGGCTCTACAC	535 bp	Initial Denaturation-94$\backslash {(}^{\circ }\text{C}$ 5m, Denaturation-94$\backslash {(}^{\circ }\text{C}$ 30s, Annealing-60$\backslash {(}^{\circ }\text{C }$30s, Extension-72$\backslash {(}^{\circ }\text{C}$ 1m; cycle 35, and Final extension-72$\backslash {(}^{\circ }\text{C}$ 7m.	[38]
	GAGCCCGCGCATCTTCAG			
*asc*V	GCCCGTTTTGCCTATCAA	807 bp	Initial Denaturation-94$\backslash {(}^{\circ }\text{C}$ 2m, Denaturation-94$\backslash {(}^{\circ }\text{C}$ 30s, Annealing-56$\backslash {(}^{\circ }\text{C }$50s, Extension-72$\backslash {(}^{\circ }\text{C}$ 30s, cycle 30, and Final extension-72$\backslash {(}^{\circ }\text{C}$ 10m. (modified)	[25]
	GCGCCGATATCGGTACCC			
*lip*	ATCTTCTCCGACTGGTTCGG	382 bp	Initial Denaturation-95$\backslash {(}^{\circ }\text{C}$ 10m, Denaturation-95$\backslash {(}^{\circ }\text{C}$ 15s, Annealing-66$\backslash {(}^{\circ }\text{C }$30s, Extension-72$\backslash {(}^{\circ }\text{C}$ 30s; cycle 25, and Final extension-72$\backslash {(}^{\circ }\text{C}$ 10m.	[36]
	CCGTGCCAGGACTGGGTCTT			
*ser*	CAC CGA AGT ATT GGG TCA GG	350 bp	Initial Denaturation-94$\backslash {(}^{\circ }\text{C}$ 2m, Denaturation-94$\backslash {(}^{\circ }\text{C}$ 30s, Annealing-56$\backslash {(}^{\circ }\text{C }$50s, Extension-72$\backslash {(}^{\circ }\text{C}$ 30s; cycle 35, and Final extension-72$\backslash {(}^{\circ }\text{C}$ 10m.	[35]
	GGC TCA TGC GTA ACT CTG GT			
*gcat*	CTCCTGGAATCCCAAGTATCAG	237 bp	Initial Denaturation-94$\backslash {(}^{\circ }\text{C}$ 2m, Denaturation-94$\backslash {(}^{\circ }\text{C}$ 30s, Annealing-64$\backslash {(}^{\circ }\text{C }$50s, Extension-72$\backslash {(}^{\circ }\text{C}$ 30s; cycle 35, and Final extension-72$\backslash {(}^{\circ }\text{C}$ 10m.	[35]
	GGCAGGTTGAACAGCAGTATCT			

**Legends:** bp: base pair; m: minutes; s: seconds; $\backslash {(}^{\circ }\text{C}:\;$degree Celsius; F: forward primer; R: reverse primer

### Antibiogram study

In this study, an antibiotic susceptibility test was carried out in order to determine the antibiotic resistance pattern to commonly used antibiotics at the field level of investigation. The antimicrobial resistance profiles of *A. hydrophila* were determined using the disc diffusion method, following the standard operating procedures, provided by the Clinical Laboratory Standards Institute (CLSI) [39]. APW broth was used to culture the isolates, which were kept at 37°C for 4 hr in a shaker incubator. It was necessary to measure the zone diameter (in millimetres) of the antibiotic discs after they were incubated for 18 hr at 37°C temperature on the inoculated surface of the Mueller Hinton agar (HI-Media, India). The 20 antibiotic discs, including Penicillin G (P; 10 µg), Amoxicillin (AML; 25 µg), Ampicillin (AMP; 10 µg) Azithromycin (AZM; 15 µg), Chloramphenicol (C; 30 µg), Florfenicol (FFC; 30 µg), Meropenem (MEM; 10 µg), Tetracycline (TE; 30 µg), Cephradine (CE; 30 µg), Ceftriaxone (CRO; 30 µg), Cefuroxime (CXM; 30 µg), Aztreonam (ATM; 30 µg), Streptomycin (S; 10 µg), Kanamycin (K; 30 µg), Gentamicin (CN; 10 µg), Cotrimoxazole (COT; 25 µg), Nalidixic acid (NA; 30 µg), Ciprofloxacin (CIP; 5 µg), Levofloxacin (LEV; 5 µg), and Erythromycin (E; 15 µg), were used in this study. All the discs were purchased from Oxoid Limited (Hampshire, UK), except COT (HiMedia Laboratories Private Ltd., Maharashtra, India). The multidrug resistance (MDR) patterns were assessed, and the multiple antibiotic resistance (MAR) index was calculated as the ratio of the number of antibiotics to which an isolate was resistant to the total number of antibiotics tested. A threshold value of 0.2 was used to identify high-risk contamination sources, as commonly cited in previous studies Magiorakos et al. [40] and Krumperman et al. [41].

### Statistical analysis

The categorical variables were analysed by using chi-square test. All data analyses were performed by SPSS software (version 25.0). A p-value <0.05 was considered statistically significant.

## Results

**Fig 2:**
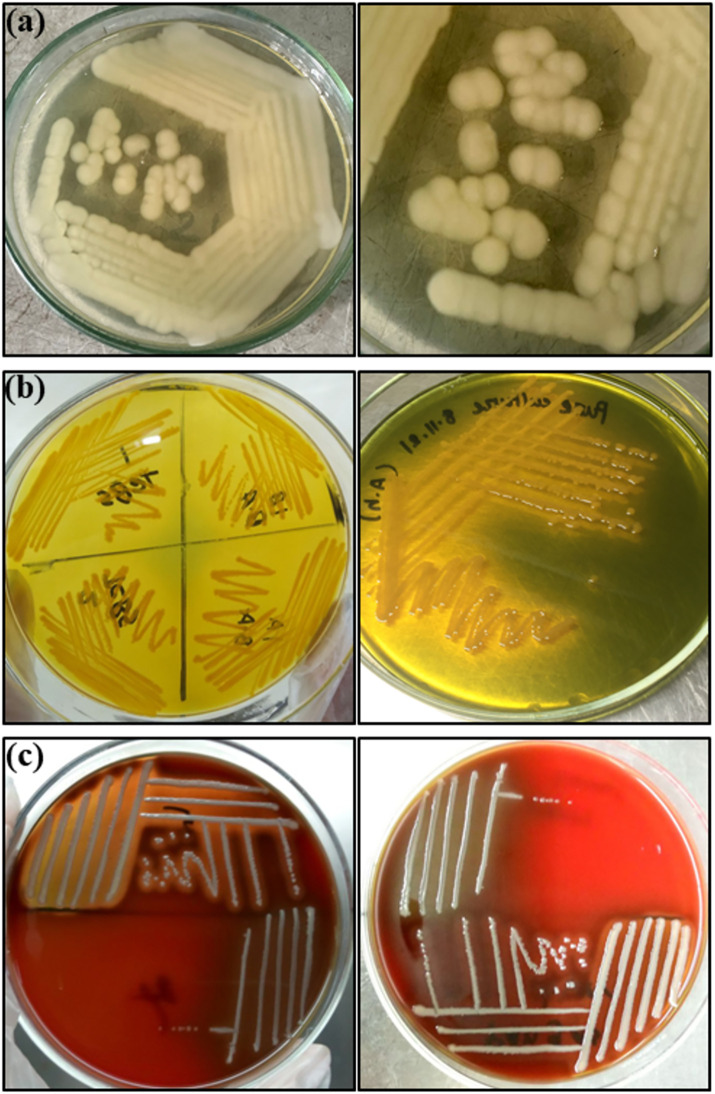
Growth of suspected *Aeromonas* spp. on different agar media. (a) TSA (light yellowish creamy smooth colonies); (b) TCBS agar (yellow shin with diameter 2-3 mm); (c) Sheep blood agar (pale white smooth colonies with *β*-haemolysis).

Out of 140 samples, a total of 38 (27.14%) were positive, with 16 (50) stinging catfish and 6 (20) shark catfish at Trishal and 12 (50) stinging catfish and 4 (20) shark catfish at Muktagachha. Bacteriological examination of all 38 positive organisms revealed yellowish opaque, round, convex, and smooth colonies on TSA and yellow shin with diameter ranged 2–3 mm on TCBS agar. Subsequently, in 5% sheep blood agar the organism showed smooth, convex, rounded *β*-haemolytic and pale white colour colonies (Fig 2). Furthermore, the motility test in MIU medium clearly demonstrated that *A. hydrophila* has a high level of motility (Table 2). The Gram’s staining of the isolates revealed as Gram-negative with straight short rod shaped under light microscope. In terms of biochemical tests, the positive isolates tested positive for catalase, VP, and indole, as well as sugar fermentation tests such as Dextrose, Maltose, Sucrose, and Mannitol, and lactose fermentation, MR test negative Table 2.

**Table 2:** Morphological and biochemical characterization of *A. hydrophila.*

Characters	Positive	Negative
*β*-hemolysis	All positive	
Motility	36 isolates	2 isolates
Gram’s staining	-ve (short rod)	
VP test	+	
Indole	+	
MR test		–
Sugar Fermentation test		
Dextrose	+	
Maltose	+	
Sucrose	+	
Mannitol	+	
Lactose	+	–

**Legends:** ‘+’ indicates positive result; ‘-’ indicates negative result.

### Molecular detection (PCR-based) of the organisms

**Fig 3:**
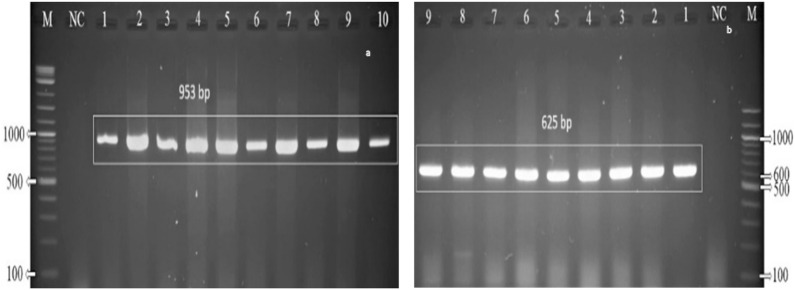
Representative picture of PCR amplification detecting *Aeromonas hydrophila.* [a] PCR amplification of genus-specific *16S rDNA* gene sequence of *A. hydrophila*; M: 1 kb molecular marker; NC: negative control; Lane 1-10: positive *Aeromonas* spp*.* at 953 bp; [b] species-specific *16S rDNA* gene-based PCR detection of *A. hydrophila*; M: 100 bp DNA ladder; NC: negative control; Lane 1-9: positive *A. hydrophila* at 625 bp amplicon size.

The phenotypically (cultural, staining, and biochemical methods based) identified *Aeromonas* spp. isolates were subjected to PCR, using genus- and species-specific primers from *16S rDNA* gene of the organism. The genus-specific primer-based PCR revealed desired and consistent positive band at 953 bp on the gel electrophoresis (Fig 3a). While further confirmation at species level, all *A. hydrophila* positive isolates depicting a 625 bp *16s rDNA* gene product size, in species-specific primer-based PCR assay (Fig 3b).

### Distribution of virulence genes of *A. hydrophila*

**Fig 4:**
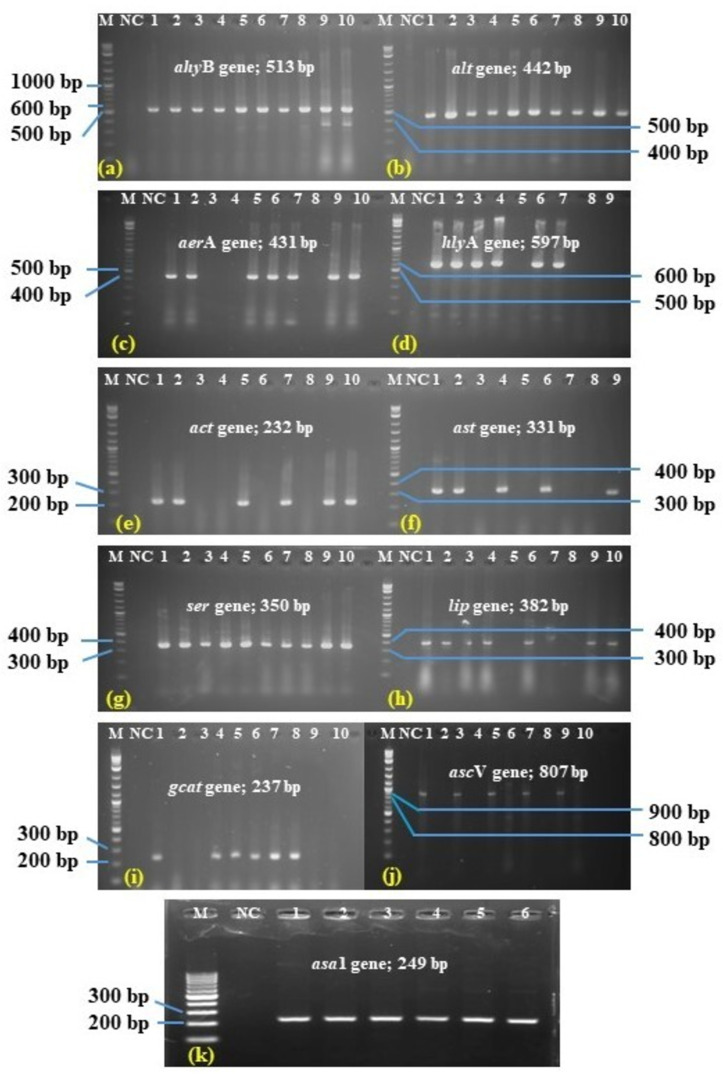
PCR amplification of virulence genes. M: 1 kb ladder; NC: negative control, [a]. elastase (*ahy*B) gene; (1-10) lane with 513 bp, [b]. heat labile enterotoxin (*alt*) gene; (1-10) lane at 442 bp, [c]. aerolysin A (*aer*A) gene; (3,4,8) lane negative and (1,2,5,6,7,9,10) lane positive with 431 bp, [d]. haemolysin A (*hly*A); lane 5,8,9 negative and (1,2,3,4,6,7) lane positive with 597 bp, [e]. cytotoxic enterotoxin (*act*); lane 3,4,6,8 negative and rest of the lane positive with 232 bp, [f]. cytotoxic heat stable enterotoxin (*ast*); lane 1,2,4,6,9 positive 331 bp, and lane 3,5,7,8 negative [g]. serine protease (*ser*); lane (1-10) positive with 350 bp, [h]. lipase (*lip*); lane 5,8 negative and rest of positive with 382 bp, [i]. glycerophospholipid-cholesterol acyl transferase (*gcat*); lane 2,3,9,10 negative and lane 1,4,5,6,7,8 positive with 237 bp, [j]. type 𝐈𝐈𝐈 secretion (*asc*V); lane 1,3,5,7,9 positive 807 bp and lane 2,4,6,8,10 negatives, [k]. haemolysin-homolog (*asa*1); lane (1-6) positive 249 bp.

**Fig 5:**
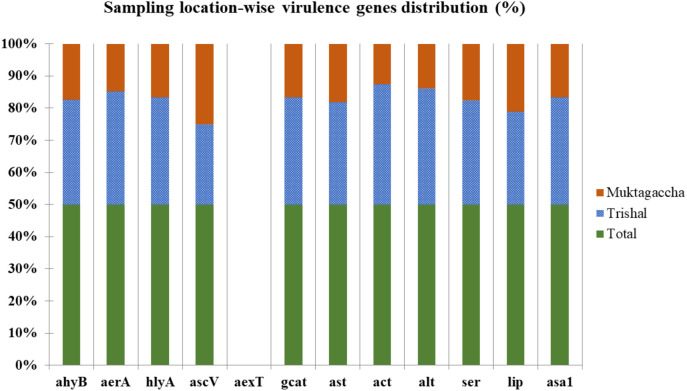
Prevalence of virulence genes in Trishal and Muktagachha.

All *16S rDNA*-positive *A. hydrophila* isolates were tested twelve (12) including *ahyB, aer*A*, hly*A*, asa*1*, asc*V*, aex*T*, gcat, ast, act, alt, ser and lip* virulence genes. Among these 12 virulence genes, the *ahy*B*, aer*A*, alt, ser* and *lip* were found most common in all isolates. The elastase (*ahy*B) gene showed the highest rate of 68.18% and 50% both for stinging catfish and shark catfish in Trishal and Muktagachha respectively. In addition, the aerolysin (*aer*A) and cytotoxic heat labile (*alt*) genes presented 54.54% and 59.09% in Trishal, 31.25% in Muktagachha. However, the lowest occurrence of type III secretion (*asc*V) and cytotoxic heat labile enterotoxin (*act*) genes were 22.72% and 27.27% in Trishal and 18.75% as well as 12.5% in Muktagachha (Table 3 and Fig 4). In case of ADP-ribosylating toxin (*aex*T), no occurrence was found. The occurrence of all virulence genes, in respective to the sampling locations, was represented in the Fig 5.

**Table 3:** Occurrence of virulence genes of *A. hydrophila* in stinging catfish and shark catfish of Trishal and Muktagachha upazila.

Total sample		Virulence genes of *Aeromonas hydrophila*											
		*ahy*B	*aer*A	*hly*A	*asc*V	*aex*T	*gcat*	*ast*	*act*	*alt*	*ser*	*lip*	*asa1*
Trishal	ST (16)	12	10	8	3	0	6	5	4	10	7	8	8
	Occurrence (%)	75	62.5	50	18.8	0	37.5	31.25	25	62.5	43.8	50	50
	PT (6)	3	2	2	1	0	0	2	2	3	4	3	2
	Occurrence (%)	50	33.33	33.33	16.66	0	0	33.33	33.33	50	66.7	50	33.33
	Total occurrence %	68.18	54.54	45.45	22.72	0	27.27	31.81	27.27	59.09	50	50	45.45
Muktagachha	SM (12)	6	4	4	4	0	2	4	2	4	4	6	4
	Occurrence (%)	50	33.3	33.3	33.3	0	16.7	33.3	16.7	33.3	33.3	50	33.3
	PM (4)	2	1	1	0	0	1	0	0	1	2	2	1
	Occurrence (%)	50	25	25	0	0	25	0	0	25	50	50	25
	Total occurrence %	50	31.25	31.25	18.75	0	18.75	25	12.5	31.25	37.5	50	31.25
	**Grand total**	**60.52**	**44.73**	**39.47**	**21.05**	**0**	**23.68**	**28.94**	**21.05**	**47.36**	**44.73**	**50**	**39.47**

Legends: ST: shing (stinging catfish) from Trishal; PT: Pangasius (shark catfish) from Trishal; SM: shing (stinging catfish) from Muktagachha; PM: Pangasius (shark catfish) from Muktagachha; Upazila: Sub-district.

### Antibiotic susceptibility of *A. hydrophila* isolates

**Fig 6:**
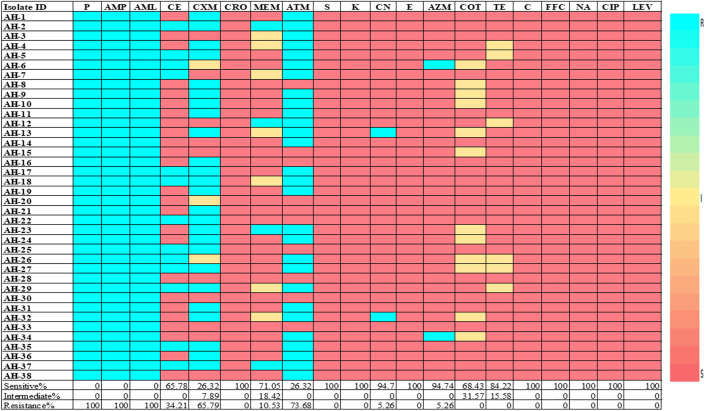
Heatmap showing antibiotic susceptibility pattern of *A. hydrophila* isolates. P: Penicillin G; AMP: Ampicillin; AML: Amoxicillin; CE: Cephradine; CXM: Cefuroxime; CRO: Ceftriaxone; MEM: Meropenem; ATM: Aztreonam; S: Streptomycin; K: Kanamycin; CN: Gentamicin, E: Erythromycin; AZM: Azithromycin; COT: Cotrimoxazole; TE: Tetracycline; C: Chloramphenicol; FFC: Florfenicol; NA: Nalidixic acid; CIP: Ciprofloxacin; LEV: Levofloxacin.

Regarding the antibiotic resistance profile of *A. hydrophila*, the results revealed that 100% isolates showed resistance to only Penicillin group of antibiotics (Penicillin-G, Amoxicillin, and Ampicillin). In contrast, 100% (38/38) isolates showed sensitivity toward β-lactam-Cephalosporin (Ceftriaxone), Aminoglycosides (Streptomycin, Kanamycin), Macrolides (Erythromycin), Phenicols (Chloramphenicol, Florfenicol), and Quinolones (Nalidixic acid, Ciprofloxacin and Levofloxacin) susceptibility to *A. hydrophila*. Again, highest variable sensitivity patterns were observed against Gentamicin and Azithromycin (94.74%), followed by Tetracycline 84.21%, Meropenem 71.05%, Cotrimoxazole 68.42%, and Cephradine 65.79%, while Cefuroxime and Aztreonam were only 26.31%. Subsequently, Cefuroxime and Aztreonam showed highest variable resistance which were 73.68% in both cases, followed by 34.21%, 31.58%, and 28.95% in case of Cephradine, Cotrimoxazole, and Meropenem, respectively. The antimicrobial class-wise detail sensitivity-resistance patterns is presented in the Table 4 and Fig 6.

**Table 4:** The antibiotic susceptibility profile of *A. hydrophila* isolates.

Antibacterial classes	Antibacterial compound		Antibacterial susceptibility (%)	
			Susceptible	Resistant
Beta lactam antibiotics				
Penicillin	Penicillin G		–	100 (38/38)
	Ampicillin		–	100 (38/38)
	Amoxicillin		–	100 (38/38)
Cephalosporin	1^st^ generation	Cephradine	65.79 (25/38)	34.21 (13/38)
	2^nd^ generation	Cefuroxime	26.32 (10/38)	73.68 (28/38)
	3^rd^ generation	Ceftriaxone	100 (38/38)	–
Penem	Meropenem		71.05 (27/38)	28.95 (11/38)
Monobactams	Aztreonam		26.32 (10/38)	73.68 (28/38)
Non-beta lactam antibiotics				
Aminoglycosides	Narrow spectrum	Streptomycin	100 (38/38)	–
	Broad spectrum	Kanamycin	100 (38/38)	–
	Extended spectrum	Gentamicin	94.74 (36/38)	5.26 (2/38)
Macrolides	Erythromycin		100 (38/38)	–
Semi-synthetic erythromycin	Azithromycin		94.74 (36/38)	5.26 (2/38)
Sulphonamides	Cotrimoxazole		68.42 (26/38)	31.58 (12/38)
Tetracyclines	Tetracycline		84.21 (32/38)	15.79 (6/38)
Phenicol	Chloramphenicol		100 (38/38)	–
	Florfenicol		100 (38/38)	–
Quinolones	1^st^ generation	Nalidixic acid	100 (38/38)	–
	2^nd^ generation	Ciprofloxacin	100 (38/38)	–
	3^rd^ generation	Levofloxacin	100 (38/38)	–

### MDR and MAR index analysis of the *A. hydrophila* isolates

Among the 38 PCR-confirmed *A. hydrophila* isolates, subjected to antibiogram profiling, multi-drug resistance (MDR) patterns (resistance to at least one antimicrobial agent of ≥ 3 antimicrobial classes) were observed in 27 (71.05%) isolates. Among MDR isolates, fish-wise MDR results revealed that 77.78% stinging catfish isolates (21/27) showed MDR patterns and 22.22% (6/27) shark catfish isolates were MDR. Again, among stinging catfish isolates of *A. hydrophila*, 75% (21/28) were MDR and 60% (6/10) shark catfish isolates were MDR. However, sampling location-wise distribution revealed somewhat variable results. Stinging catfish isolates from Trishal and Muktagachha regions showed 81.25% (13/16) and 66.67% (8/12) MDR patterns, respectively, whereas, shark catfish isolates from Trishal and Muktagachha regions revealed 50% (3/6) and 75% (3/4) MDR patterns, respectively. While analysing multiple-antibiotic resistance (MAR) index, the highest MARI value was found in 6 isolates (MARI 0.40; 15.79%; Sample ID: AH-6, AH-13, AH-26, AH-27, AH-29, and AH-32) and lowest in 3 isolates (MARI 0.15; 7.89%; Sample ID: AH-28, AH-30, AH-33). A total of 35 isolates (92.11%) had shown the MARI values of ≥0.2, moreover, 6 (15.79%), 8 (21.05%), 9 (23.68%), and 6 (15.79%) isolates had the MARI values of 0.35, 0.30, 0.25, and 0.20, respectively. The detail of the MDR and MAR index of all 38 isolates is depicted in the Table 5.

**Table 5:** Isolate-wise MDR, MAR index analysis and virulence gene distribution of the *A. hydrophila* isolates.

Isolate ID	Fish ID	Antibiotic Resistance patterns	No. of Antibiotics resistant (classes)	MAR Index	Virulence gene
AH-1	ST4	P, AMP, AML, CXM, ATM	5(3)	0.25	*ahy*B*, hly*A*, gcat, act, lip*
AH-2	ST10	P, AMP, AML, CE, CXM, MEM, ATM	7(4)	0.35	*ahy*B*, aer*A*, ast, lip, asa*1
AH-3	ST16	P, AMP, AML, MEM, ATM	5(3)	0.25	*aer*A*, hly*A*, gcat, alt, ser, asa*1
AH-4	ST17	P, AMP, AML, CXM, MEM, ATM, TE	7(5)	0.35	*ahy*B*, asc*V*, ast, alt, lip*
AH-5	ST20	P, AMP, AML, CE, CXM, ATM, TE	7(4)	0.35	*ahy*B*, aer*A*, hly*A*, gcat, asa*1
AH-6	ST22	P, AMP, AML, CE, CXM, ATM, AZM, COT	8(5)	0.40	*ahy*B*, aer*A*, hly, act, alt, ser, lip*
AH-7	ST26	P, AMP, AML, CE, MEM, ATM	6(4)	0.30	*ahy*B*, aer*A*, ast, ser, asa*1
AH-8	ST27	P, AMP, AML, CXM, COT	5(3)	0.25	*ahy*B*, aer*A*, gcat, alt, asa*1
AH-9	ST28	P, AMP, AML, CXM, ATM, COT	6(4)	0.30	*aer*A*, hly*A*, alt*
AH-10	ST30	P, AMP, AML, CXM, ATM, COT	6(4)	0.30	*asc*V*, gcat, act, alt, lip*
AH-11	ST33	P, AMP, AML, CXM, ATM	5(3)	0.25	*ahy*B*, aer*A*, hly*A*, ast, ser*
AH-12	ST37	P, AMP, AML, MEM, ATM, TE	6(4)	0.30	*hly*A*, asc*V*, alt, ser, lip, asa*1
AH-13	ST42	P, AMP, AML, CXM, MEM, ATM, CN, COT	8(6)	0.40	*ahy*B*, aer*A*, alt, asa*1
AH-14	ST43	P, AMP, AML, ATM	4(2)	0.20	*ahy*B*, gcat, act, ser, lip*
AH-15	ST45	P, AMP, AML, COT	4(2)	0.20	*ahyB, aer*A*, ast, alt, lip, asa*1
AH-16	ST48	P, AMP, AML, CXM	4(2)	0.20	*ahy*B*, hly*A*, alt, ser*
AH-17	PT1	P, AMP, AML, CE, MEM, CXM, ATM	7(4)	0.35	*ahy*B*, alt, ser, lip*
AH-18	PT6	P, AMP, AML, CE, CXM, ATM	6(3)	0.30	*ast, act, ser, asa*1
AH-19	PT11	P, AMP, AML, CXM, ATM	5(3)	0.25	*hly*A*, asc*V*, alt, lip*
AH-20	PT14	P, AMP, AML, CXM	4(2)	0.20	*aer*A*, hly*A*, ast, ser*
AH-21	PT18	P, AMP, AML, CXM	4(2)	0.20	*ahy*B*, aer*A*, alt, asa*1
AH-22	PT19	P, AMP, AML, CE, CXM	5(2)	0.25	*ahy*B*, act, ser, lip*
AH-23	SM1	P, AMP, AML, CXM, MEM, ATM, COT	7(5)	0.35	*hly*A*, ast, alt, asa*1
AH-24	SM10	P, AMP, AML, CXM, ATM, COT	6(4)	0.30	*hly*A*, gcat, ser, lip*
AH-25	SM12	P, AMP, AML, CE, CXM	5(2)	0.25	*ahy*B*, asc*V*, ser,*
AH-26	SM13	P, AMP, AML, CE, CXM, ATM, COT, TE	8(5)	0.40	*ahy*B*, asc*V*, alt, lip*
AH-27	SM18	P, AMP, AML, CE, CXM, ATM, COT, TE	8(5)	0.40	*aer*A*, asc*V, *ast, act, asa*1
AH-28	SM20	P, AMP, AML	3(1)	0.15	*ahy*B*, alt, ser, lip*
AH-29	SM27	P, AMP, AML, CE, MEM, CXM, ATM, TE	8(5)	0.40	*ahy*B*, aer*A*, ast, lip*
AH-30	SM28	P, AMP, AML	3(1)	0.15	*ahy*B*, gcat, asa*1
AH-31	SM39	P, AMP, AML, CXM, ATM	5(3)	0.25	*hly*A*, ser, lip*
AH-32	SM43	P, AMP, AML, MEM, CXM, ATM, CN, COT	8(6)	0.40	*asc*V*, ast, alt, asa*1
AH-33	SM47	P, AMP, AML	3(1)	0.15	*aer*A*, hly*A*, lip*
AH-34	SM49	P, AMP, AML, ATM, AZM, COT	6(4)	0.30	*ahy*B*, aer*A*, act*
AH-35	PM6	P, AMP, AML, CE, CXM, ATM	6(3)	0.30	*ahy*B*, ser*
AH-36	PM12	P, AMP, AML, CXM, ATM	5(3)	0.25	*aer*A*, alt, lip*
AH-37	PM17	P, AMP, AML, CE, CXM, MEM, ATM	7(4)	0.35	*gcat, lip, asa*1
AH-38	PM20	P, AMP, AML, ATM	4(2)	0.20	*ahy*B*, hly*A*, ser*

P: Penicillin G; AMP: Ampicillin; AML: Amoxicillin; CE: Cephradine; CXM: Cefuroxime; CRO: Ceftriaxone; MEM: Meropenem; ATM: Aztreonam; S: Streptomycin; K: Kanamycin; CN: Gentamicin, E: Erythromycin; AZM: Azithromycin; COT: Cotrimoxazole; TE: Tetracycline; C: Chloramphenicol; FFC: Florfenicol; NA: Nalidixic acid; CIP: Ciprofloxacin; LEV: Levofloxacin; AH: *Aeromonas hydrophila*; ST: shing fish (stinging catfish) from Trishal; PT: pangasius (shark catfish) from Trishal; SM: shing fish from Muktagachha; PM: pangasius from Muktagachha; MDR: multi-drug resistant; MAR index: multiple antibiotic resistance index.

### Relationship between phenotypic resistance and virulence associated genes in *A. hydrophila* isolates

The phenotypic resistance patterns and the presence of virulence associated genes of the *A. hydrophila* isolates were further analysed to assess whether any relationship exist between phenotypic resistance and virulence genes, which were expressed statistically using Chi-square test, among antibiotic susceptible and non-susceptible (resistant) isolates (Tables 6 and 7). It was found that the presence of cytotoxic enterotoxin gene (*act*) showed highly significant association (p-value 0.005) with the phenotypic resistance of the isolates against Azithromycin (AZM). Similarly, the presence of one of the members of the TTSS (*asc*V gene-encoded) was also highly significant with the phenotypic resistant isolates against Tetracycline (p-value 0.003). Interestingly, phenotypically resistance isolates against Meropenem and Cotrimoxazole showed more prominent features of association. Meropenem resistant isolates revealed highly significant association with the presence of haemolysin-homolog *asa*1 (p-value 0.007) and *ast* gene encoded cytotoxic stable toxin (p-value 0.003). Similarly, Cotrimoxazole resistant isolates demonstrated the highly significant association with the presence of *alt* gene-encoded cytotoxic labile toxin (p-value 0.037) and *ser* gene-encoded serine protease (p-value 0.009).

**Table 6:** Association between phenotypic resistance and virulence factors (haemolysin and cytotoxic type) in *A. hydrophila* isolates.

Antibiotic(s)	Prevalence (%) of virulence genes																	
	*aer*A			*hly*A			*asa*1			*act*			*ast*			*alt*		
	NSI	SI	P-value	NSI	SI	P-value	NSI	SI	P-value	NSI	SI	P-value	NSI	SI	P-value	NSI	SI	P-value
P	44.7	0	–	39.5	0	–	39.5	0	–	21.1	0	–	28.9	0	–	47.4	0	
AML	44.7	0	–	39.5	0	–	39.5	0	–	21.1	0	–	28.9	0	–	47.4	0	
AMP	44.7	0	–	39.5	0	–	39.5	0	–	21.1	0	–	28.9	0	–	47.4	0	
AZM	100	41.7	.106	50	38.9	.754	0	41.7	.241	100	16.7	.005*	0	30.6	.354	50	47.2	.939
C	0	44.7		0	39.5	–	0	39.5	–	0	21.1	–	0	28.9	–	0	47.4	
FFC	0	44.7	.508	0	39.5	–	0	39.5	–	0	21.1	–	0	28.9	–	0	47.4	
MEM	36.4	48.1	–	27.3	44.4	.326	72.7	25.9	.007*	9.1	25.9	.248	63.6	14.8	.003*	63.6	40.7	.200
TE	50	43.8	.778	33.3	40.6	.737	50	37.5	.565	16.7	21.9	.774	50	25	.215	50	46.9	.888
CE	46.2	44	.899	23.1	48	.136	46.2	36	.544	30.8	16	.289	38.5	24	.351	30.8	56	.139
CRO	0	44.7	.726	0	36.8	–	0	39.5	–	0	18.4	–	0	29	–	0	47.4	
CXM	32.1	20	–	39.3	40	.968	37.5	50	.428	21.4	20	.924	46.4	40	.467	50	40	.587
ATM	39.3	60	.258	46.4	20	.142	39.3	40	.968	25	10	.318	32.1	20	.467	50	40	.587
S	0	44.7	–	0	39.5	–	0	39.5	–	0	21.1	–	0	28.9	–	0	47.4	
K	0	44.7	–	0	39.5	–	0	39.5	–	0	21.1	–	0	28.9	–	0	47.4	
COT	58.3	38.5	.252	33.3	42.3	.599	41.7	38.5	.851	33.3	15.4	.207	33.3	26.9	.685	66.7	34.6	.037*
NA	0	44.7	–	0	39.5	–	0	39.5	–	0	21.1	–	0	28.9	–	0	47.4	
CIP	0	44.7	–	0	39.5	–	0	39.5	–	0	21.1	–	0	28.9	–	0	47.4	
LEV	0	44.7	–	0	39.5	–	0	39.5	–	0	21.1	–	0	28.9	–	0	47.4	
CN	50	44.4	.858	0	41.7	.241	50	38.9	.754	0	22.2	.453	50	27.8	.500	100	44.4	.126
E	0	44.7	–	0	39.5	–	0	39.5	–	0	21.1	–	0	28.9	–	0	47.4	

P: Penicillin G; AMP: Ampicillin; AML: Amoxicillin; CE: Cephradine; CXM: Cefuroxime; CRO: Ceftriaxone; MEM: Meropenem; ATM: Aztreonam; S: Streptomycin; K: Kanamycin; CN: Gentamicin, E: Erythromycin; AZM: Azithromycin; COT: Cotrimoxazole; TE: Tetracycline; C: Chloramphenicol; FFC: Florfenicol; NA: Nalidixic acid; CIP: Ciprofloxacin; LEV: Levofloxacin; NSI: non-susceptible isolates; SI: susceptible isolates.

**Table 7:** Association between phenotypic resistance and virulence factors (responsible for tissue destruction and invasion) in *A. hydrophila* isolates.

Antibiotic(s)	Prevalence (%) of virulence genes														
	*ser*			*ahy*B			*lip*			*gcat*			*asc*V		
	NSI	SI	P-value	NSI	SI	P-value	NSI	SI	P-value	NSI	SI	P-value	NSI	SI	P-value
P	44.7	0		60.5	0	–	50	0	–	23.7	0	–	21.1	0	–
AML	44.7	0		60.5	0	–	50	0	–	23.7	0	–	21.1	0	–
AMP	44.7	0		60.5	0	–	50	0	–	23.7	0	–	21.1	0	–
AZM	0	47.2	.191	100	58.3	.241	50	50	1.00	0	25	.418	0	22.2	.453
C	0	44.7		0	60.5	–	0	50	–	0	23.7	–	0	21.1	–
FFC	0	44.7		0	60.5	–	0	50	–	0	23.7	–	0	21.1	–
MEM	54.5	40.7	.438	45.5	67.7	.225	45.5	51.9	.721	18.2	25.9	.611	36.4	14.8	.139
TE	33.3	46.9	.540	66.7	59.4	.737	66.7	46.9	.374	16.7	25	.660	66.7	12.5	.003*
CE	38.5	48	.575	76.9	52	.136	53.8	48	.732	15.4	28	.386	23.1	20	.825
CRO	0	44.7		0	60.5	.475	0	50	–	0	23.7	–	0	21	–
CXM	42.9	50	.697	25	10	–	50	50	1.00	21.4	30	.584	57.1	70	.318
uATM	42.9	50	.697	53.6	80	.142	53.6	40	.461	25	20	.750	25	10	.318
S	0	44.7		0	60.5	–	0	50	–	0	23.7	–	0	21.1	–
K	0	44.7		0	60.5	–	0	50	–	0	23.7	–	0	21.1	–
COT	8.3	57.7	.009*	50	65.4	.367	41.7	53.8	.485	25	23.1	.897	25	19.2	.685
NA	0	44.7		0	60.5	–	0	50	–	0	23.7	–	0	21.1	–
CIP	0	44.7		0	60.5	–	0	50	–	0	23.7	–	0	21.1	–
LEV	0	44.7		0	60.5	–	0	50	–	0	23.7	–	0	21.1	–
CN	50	44.7	.878	50	61.1	.745	0	52.8	.146	0	25	.418	50	19.4	.302
E	0	44.7		0	60.5	–	0	50	–	0	23.7	–	0	21.1	–

P: Penicillin G; AMP: Ampicillin; AML: Amoxicillin; CE: Cephradine; CXM: Cefuroxime; CRO: Ceftriaxone; MEM: Meropenem; ATM: Aztreonam; S: Streptomycin; K: Kanamycin; CN: Gentamicin, E: Erythromycin; AZM: Azithromycin; COT: Cotrimoxazole; TE: Tetracycline; C: Chloramphenicol; FFC: Florfenicol; NA: Nalidixic acid; CIP: Ciprofloxacin; LEV: Levofloxacin; NSI: non-susceptible isolates; SI: susceptible isolates.

## Discussion

One of the principle restraints in the culture of many aquatic species is infection and this hinders the social and economic expansions in aquaculture trade and production [42]. Several *Aeromonas* spp*.,* have been implicated for causing fish diseases recently [25,43,44], however, *A. hydrophila* is causing more damage to the aquatic species which causes haemorrhage mainly to the fishes reared in the farm conditions [9,45]. It is recorded that two groups of aeromonads: mesophilic (motile *A. hydrophila*) and psychrophilic (non-motile *A. salmonicida*) that grow well at 35–37°C and 22–25°C [11,44]. Nahar et al. [7] reported various clinical features of *A. hydrophila* in catfishes, carps, and perches. The features were more or less commonly as fin rot, distended abdomen due to ascites, bilateral exophthalmia, scale bulging, epithelium ulceration, haemorrhages and petechiation, and in many cases, swollen and haemorrhagic gill lamellae, even sloughing off of skin. In the present study, typical MAS-like clinical features were found in stinging catfish and pangasius, such as loss of normal appearance, pale body colour but reddish head and anal region, ulcerative lesions on the external body surface, *viz*., haemorrhage, skin erosion and reddened fin bases, and irregular swarming, which were consistent with the previous findings [7]. The present study could be considered as the pioneer work to isolate *A. hydrophila* caused ulcerative and haemorrhagic lesions in infected aquatic species in Bangladesh.

The collected samples were enriched in APW for 24 hr at 37$\backslash {(}^{\circ }\text{C}$ primarily and Millership and Chattopadhyay [46] mentioned APW as the best recovery and enrichment medium for *A. hydrophila*. On TSA agar the suspected isolates produced yellowish opaque, round, smooth colonies which was similar to the findings of Nahar et al. [7]. Colonies on TSA agar were then streak on 5% sheep blood agar and TCBS agar plates for further confirmation. The results showed *β*- haemolytic pale white (slightly greyish) colour smooth colonies on blood agar and bright yellow colonies on TCBS agar plates. The results of cultural characterization of suspected *Aeromonas* isolates were in agreement with the previous findings of Nahar et al. [7], and Hossain et al. [24], where they examined various other media for isolation of *A. hydrophila*. The Gram’s staining and biochemical test revealed that the suspected *A. hydrophila* isolates were Gram-negative short rods, catalase positive, capable of fermenting sugar with the formation of acid and gas, VP and Indole positive but negative to methyl red. The biochemical results in this study were found consistent and in agreement with the earlier reports [5,7]. PCR results revealed that finally 38 out of 140 isolated were confirmed as *A. hydrophila*.

The incidence of *A. hydrophila* of the present study was 27.14% which was consistent with the findings of El-Bahar et al. [47] and Zhang et al. [48], who reported prevalence of *A. hydrophila* from fish samples 27.5% and 24.6%, respectively. However, the prevalence reports of *A. hydrophila* was found variable in various published articles. Ayoub et al. [49], and Monir et al. [42] reported prevalence of *A. hydrophila* as 31.6% and 40%, respectively. On the other hand, El-Hossary et al. [50] found only 3.2% as *Aeromonas* spp. and 1.2% as *A. hydrophila* from a total 343 samples, comprised of *Oreochromis niloticus*, *Mugil cephalus*, and human skin swabs and faecal samples in Egypt. Moreover, Mahmood et al. [8] found 6.46% and 6.25% *A. hydrophila* from *Channa marulius* and *Sperata sarwari* in Pakistan. This study indicates that *A. hydrophila* is most prevalent in Trishal upazila than Muktagachha upazila. This difference could be the accumulated results of geographical location, fish culture system, and the hygiene and sanitary practices of the fish farm personnel [49].

*Aeromonas* pathogenicity is complex and multi-faceted, and there is no definitive link between toxin genes and clinical presentation [9]. Different virulence genes in isolated *A. hydrophila* were investigated, such as *aer*A (aerolysin), *hly*A (haemolysin), *asa*1 (homologous to *aer* gene product), *alt* (heat-labile cytotonic enterotoxin), *act* (cytotoxic enterotoxin), *ast* (heat-stable cytotonic enterotoxin), *ahy*B (elastase), *aex*T (ADP-ribosylating Toxin), *ser* (serine protease), *lip* (lipase), *asc*V (type III secretion), and *gca*t (glycerophospholipid-cholesterol acyl transferase). In clinical isolates of *A. hydrophila*, the clonal origin of virulence factors is evidenced by the abundance and high frequency of synthesizing numerous virulence factors which might have great impact on the pathobiology of the organism [23]; however, several reports suggested as not completely accurate [51].

In the present study, 54.54% in Trishal and 31.25% in Muktagachha isolates were found positive for *aer*A gene amplification, and among the virulence associated genes, 45.45% and 31.25% for *hyl*A gene in both places respectively. El-Hossary et al. [50] also reported similar or close findings, where *aer*A and *hly*A gene occurrence were 50% and 25%, respectively. The percentage of occurrence may vary up to 100% [52,53], however, Ayoub et al. [49] reported *aer*A 57.9%, which were in close agreement with the present study, but *hly*A detection percentage was only 7.9%. The presence of *aer*A and *hly*A genes strongly proved the virulent nature of pathogenic *A. hydrophila* isolates. Moreover, aerolysin is considered as the most prevalent gene among various virulence genes in *A. hydrophila* [22,26], and could be considered as suitable molecular marker for genus- and species-specific detection of the organism [54,55]. Aerolysin exerts its effects primarily as pore-forming exotoxin, thereby establishing the organisms, and subsequently maintain the systemic infections through other various activities, such as haemolysis, enterotoxicity, and cytotoxicity [31,56]. Moreover, haemolysin (*hly*A) destroys RBC membranes, causing haemolysis and anaemia [18]. Wong et al. [19] suggested the attenuation of both *aer*A and *hly*A assist to cross off the pathogenicity of *Aeromonas* spp. [57]. Another heat-labile extracellular cytotoxic toxin is *asa*. This cytotoxic toxin (45.45% and 31.25% incidence in the present study) produced synergistic effect with aerolysin and haemolysin [23].

The genes encoding elastase (*ahy*B gene product) was identified at the highest incidence rate 68.18% and 50% in respective of two places of *A. hydrophila* isolates. Cascón et al. [20] stated elastase as essential virulence factor for pathogenic *Aeromonas* isolates, and causes alteration of the host’s cytoplasmic membrane structures, subsequently, bacterial colonization in the host tissues, results in necrosis. However, Nawaz et al. [35] reported that elastase performs its full function only in the presence of aerolysin. *In-vitro* toxicity analysis strongly revealed the potential relationship of elastase with the aerolysin in cellular damages [58]. The *ahy*B gene product contribute significantly most to the bacterium’s elastolytic activity, which is important for invasion and infection establishment [20].

Various researchers [59–61] characterized three enterotoxins at molecular level from clinical *A. hydrophila* isolates. The enterotoxins are: (1) cytotoxic enterotoxin (*act* gene product), (2) heat labile cytotonic enterotoxins (*alt* gene product; labile at 56°C), and (3) heat stable cytotonic enterotoxin (*ast* gene product; stable at 56°C) [59,60]. The *act* plays role in enhancing pathogenicity of *Aeromonas* spp. which is similar to *aer*A gene (pore formation, haemolysis, enterotoxicity, and cytotoxicity) and has the ability to cause 100% cell lysis [62,63]. According to recent findings, act causes tissue damage and an increase in fluid secretion in macrophages and IEC-6 cell line (intestinal epithelial cell line from rat) [61]. In our study, the incidence of *act* gene was not significant which was only 27.27% and 12.5% in Trishal and Muktagachha, respectively. The *ast* (28.94%) and *alt* (47.36%) were found, which are indicative to enterotoxic capability of the *A. hydrophila* isolates [26]. Previous research findings demonstrated that enterotoxins, particularly, cytotoxic enterotoxins (*act*) and heat-resistant cytotoxin (*ast*) are responsible for the development of diarrhoea [61]. Furthermore, the haemolytic activity of cytotoxic enterotoxin (*act* gene) is encoded by a different gene than that of aerolysin and haemolysin [64].

Regarding the Type III secretion system (T3SS), various researchers illustrated the role of T3SS in the pathogenesis of bacterial infection, and considered as strong virulence marker [38]. In this study, among the T3SS dependent ADP-ribosylating toxins, only *asc*V and *aex*T have been investigated. It was found that 21.05% (22.72% in Trishal and 18.75% in Muktagachha) lower rate of *asc*V gene harboured in *A. hydrophila*. Aguilera-Arreola et al. [65] reported almost similar findings (25.80%) from fishes, sea water, vegetables, and meat products. However, Senderovich et al. [66] prevalence rate of only 12% from human diarrhoeic patients. The *asc*V gene was also reported from fresh water fishes [38,67,68]. Interestingly, no positive PCR band was revealed for *aex*T (ADP-ribosylating toxin) gene. In fact, the *aex*T gene prevalence has been found in lowest rate by Aguilera-Arreola et al. [65] and Senderovich et al. [66], where the authors reported only 8% and 6%, respectively, much lower than the other virulence associated genes.

The other virulence factors, investigated in the present study, such as serine protease (*ser*), lipase (*lip*), and glycerophospholipid-cholesterol acyl transferase (*gca*t), have been shown to work together to influence the pathobiology and clinical features of *Aeromonas* infections [69]. Lipase acts as an extracellular protein and alters the host plasma membrane, thereby aggravate the severity of clinical manifestations [69]. The overall, incidence of *ser* (serine protease) and *lip* (lipase) were 44.73% and 50% in this study which support other studies.

In fact, antimicrobial resistance (AMR) is a global public health concerns, however, antimicrobial applications are still the cost effective mostly used methods to control infectious disease outbreaks [27]. Antibiogram profile of the isolated *A. hydrophila* from fish samples was performed in this present study. The results revealed that 100% isolates showed resistance to Penicillin group of antibiotics (Penicillin-G, Amoxicillin, and Ampicillin). Almost similar result that *Aeromonas* strains were resistant to β-lactams antibiotics, particularly, Ampicillin and Amoxicillin, were reported by various researchers [8,49]. Like most of the Gram-negative bacteria, the genus *Aeromonas* shows natural resistance to β-lactams antibiotics [28]. Subsequently, other β-lactams such as (Cephradin, Cefuroxime), Monobactam (Aztreonam) presented 34.21%, 73.68% and 73.68% resistance, respectively, while Aminoglycoside (Gentamicin) and semi-synthetic Erythromycin (Azithromycin) were found 5.26% resistant. The 28.95% isolates showed resistance to Meropenem, as previous researchers reported resistant *A. hydrophila* isolates from cat fish and water environment [70]. The authors reported 4.3% carbapenemase producing *A. hydrophila* isolates from rainbow trout. Moreover, the human clinical isolates of *A. hydrophila* are more frequently resistant to carbapenem [71]. Roges et al. [12] also reported 13.7% resistant *A. hydrophila* (isolated from animal, food, and human sources in Brazil) to Imipenem (Carbapenem). In contrast, the Aminoglycosides (Streptomycin, Kanamycin), Macrolides (Erythromycin), Phenicols (Chloramphenicol, Florfenicol), Quinolones (Nalidixic acid, Ciprofloxacin and Levofloxacin) and β-lactam (3^rd^ generation Cephalosporin; Ceftriaxone) illustrated 100% susceptibility to *A. hydrophila*. According to Zdanowicz et al. [72] Ciprofloxacin and Chloramphenicol showed very low percentage of resistance to *Aeromonas* isolates from ponds. In addition, Gentamycin and Azithromycin were found more effective 94.74%, while Cefuroxime and Aztreonam were only 26.32%. The same observations were explained by Zdanowicz et al. [72].

The detection of multidrug-resistant (MDR) *A. hydrophila* in aquaculture-raised fish raises significant public health concerns, particularly regarding its potential role in the dissemination of antimicrobial resistance (AMR) through the food chain [73]. Fish and aquatic environments can act as reservoirs and vectors for antimicrobial resistance genes (ARGs), which may be transmitted to humans through the handling or consumption of contaminated fish products, or via water used in aquaculture systems [29]. Regarding the MDR patterns, in the present study, 71.05% isolates (regardless of fish type and locations) were found as MDR. Various researchers also reported MDR *A. hydrophila* isolates from different sources, even extremely drug resistant (XDR) isolates [17,49]. Lee et al. [16] reported 58% MDR *Aeromonas* spp. isolates from ready-to-eat foods in Norway. Eid et al. [17] observed 90% MDR and 26.6% XDR *Aeromonas* spp. from wild *Mugil cephalus* (stripped mullet) and Mediterranean seawater, where the *A. hydrophila* was the predominant species. In Egypt, El-Hossary et al. [50] and Ayoub et al. [49] found 50% and 63.1% MDR *A. hydrophila* isolates, respectively, from different tilapia species and *Mugil cephalus*, including human hand swab and stools samples. In Pakistan, Mahmood et al. [8] reported 100% isolates of *A. hydrophila* as MDR in *Channa marulius* and *Sperata sarwari* fish samples from rivers of Punjab. However, low percentage of MDR isolates was also reported, such as, Rao et al. [28] reported only 6.90% MDR *Aeromonas* spp. from mussel and oyster shellstock in Canada.

Again, multiple antibiotic resistance (MAR) index values found, in this study, varied from 0.15 to 0.40 for the isolates. El-Hossary et al. [50] reported MARI values of *A. hydrophila* within the range of 0.285 to 0.642, found in tilapia and stripped mullet in Egypt, however, Ayoub et al. [49] reported MARI values with broader range of 0.16 to 0.83, from the similar fish types and within the same country. The present findings also revealed the isolates as the high-risk source of contamination [41,74]. Beside the indiscriminate use of antimicrobials, the residual effect in environment, particularly waterbodies, might play a significant role in surging AMR/MDR bacterial isolates in the nature [27]. The phenotypic antimicrobial resistance in *A. hydrophila* is a complicated process as of other bacteria, emerged from simultaneous and interactive role of various factors of epidemiological triad [28,49]. This complexity may involve horizontal gene transfer, plasmid-mediated resistance, integrons, and other mobile genetic elements, which facilitate the dissemination of both antimicrobial resistance and virulence genes among bacterial populations. Again, the non-pathogenic and opportunistic pathogens might contribute to AMR bacterial emergence [28].

In the present study, the possible association between antibiotic resistance or susceptibility patterns and presence of virulence factors had also been assessed. It was found statistically that there were few highly significant associations within the resistance patterns and virulence factors, such as, AZM<+>*act*, TE<+>*asc*V, MEM<+>*asa*1, MEM<+>*ast*, COT<+>*alt*, COT<+>*ser*. In fact, the regulation and expression of genes responsible for antibiotic resistance and virulence for the bacteria are complex and interconnected, moreover, are influences by various host-related and environmental factors [75]. There are both positive and negative correlation between these antimicrobial resistance and virulence, as reported by many researchers [76,77], moreover, genes for both factors reside on mobile genetic elements, and are sometimes, coregulated [78]. In case of *Pseudomonas*, the prevalence of the virulence gene, *exo*U, is proportionately associated with the fluoroquinolone-resistance [79]. Similarly, *bla*_CTX-M15_ positive isolates of uropathogenic *E. coli* (UPEC) demonstrated higher carriage of *col*V, *hly*A, and *csg*A genes, whereas, quinolone-resistant UPEC demonstrated decreased type-1 fimbriae expression [80,81]. Most importantly, Govender et al. [82] reported the highly significant positive association between the *aer* gene with the *bla*_OXA_ and ceftazidime (P < 0.05). However, all these reports were laboratory based, or statistical analysis based, therefore, need to be addressed at molecular level and host- or laboratory animal-based pathogenicity model tests.

Future studies should incorporate molecular techniques such as whole-genome sequencing (WGS), comparative genomics, and plasmid profiling to investigate whether virulence and resistance genes are co-located on mobile genetic elements, such as integrons or plasmids. Such molecular research is essential to validate and explain the observed statistical associations between AMR and virulence in *A. hydrophila*.

It is important to acknowledge that all fish samples in this study were collected from two upazilas Trishal and Muktagachha within the Mymensingh Division of Bangladesh. While these locations are representative of common aquaculture practices in the region, the geographic restriction may limit the generalizability of our findings to other areas with different environmental, farming, or antimicrobial usage patterns. Furthermore, the sample size (n = 140) may constrain the statistical power of the study, particularly for detecting rare virulence genes or low-prevalence antimicrobial resistance patterns. In addition, the study relied on conventional PCR methods targeting selected genes; however, whole-genome-based approaches could offer deeper insights into the evolutionary mechanisms, genetic diversity, and dissemination pathways of AMR and virulence determinants. Therefore, future investigations should consider incorporating high-throughput sequencing technologies for a more comprehensive understanding of *A. hydrophila* epidemiology in Bangladesh. Broader surveillance involving larger sample sizes across multiple regions of Bangladesh would provide a more comprehensive understanding of the distribution and public health risk of *Aeromonas hydrophila* in aquaculture.

## Conclusions

From the present study, it could be concluded that pathogenic *Aeromonas hydrophila* is prevalent in the study areas (Trishal and Muktagachha upazila under Mymensingh district, Bangladesh), having numerous virulence factors with varying degree of antibiotic susceptibility. The virulence profile and its diversity as well as the antibiotic resistance of *A. hydrophila* clearly indicated that the prevalent isolates could be considered as serious threat to aquaculture sustainability and public health. This research could be the very first report of molecular detection, virulence profile, and antibiogram of *A. hydrophila* from stinging catfish and shark catfish in Bangladesh, so far. In light of these findings, AMR surveillance in Bangladeshi aquaculture is urgently needed to mitigate potential public health risks and ensure sustainable fish farming.
